# Intramedullary Spinal Cord Metastasis Mimicking Astrocytoma: A Rare Case Report

**DOI:** 10.3390/brainsci11091124

**Published:** 2021-08-25

**Authors:** Pierfrancesco Lapolla, Placido Bruzzaniti, Leopoldo Costarelli, Alessandro Frati, Rui Chen, Xiaobo Li, Selenia Miglietta, Giuseppe Familiari, Pietro Familiari

**Affiliations:** 1 Department of Anatomy, Histology, Forensic Medicine and Orthopedics, Sapienza University of Rome, 00161 Rome, Italy; selenia.miglietta@uniroma1.it (S.M.); giuseppe.familiari@uniroma1.it (G.F.); 2 Department of Human Neurosciences, Division of Neurosurgery, Policlinico Umberto I University Hospital, Sapienza, University of Rome, 00161 Rome, Italy; placido.bruzzaniti@uniroma1.it (P.B.); pietro.familiari@uniroma1.it (P.F.); 3 Department of Pathology, San Giovanni-Addolorata Hospital, 00184 Rome, Italy; lcostarelli@hsangiovanni.roma.it; 4 Department of Neurosurgery, Istituto di Ricovero e Cura a Carattere Scientifico (IRCCS) Neuromed, 86077 Pozzilli, Italy; alessandro.frati@uniroma1.it; 5 Department of Environmental Medicine and Toxicology, Southeast University, Nanjing 210096, China; ruichen@ccmu.edu.cn (R.C.); 101011116@seu.edu.cn (X.L.)

**Keywords:** intramedullary metastasis, carcinoid tumour, neuroendocrine lung tumour, spinal cord metastasis, small-cell neuroendocrine carcinoma

## Abstract

Intramedullary spinal cord metastases (ISCMs) are infrequent lesions. Their incidence is estimated to range from 0.9 to 2.1%, found in autopsies of cancer patients. However, as the life expectancy of malignant tumour patients constantly increases, the reported incidences of ISCMs are consequently rising. This report presents a case of the misdiagnosis of an anaplastic astrocytoma type of tumour due to its similarities to small-cell neuroendocrine carcinoma. Therefore, we would like to underline the importance of further investigation that could assist and support the surgeon in the making of the differential diagnosis. We present the clinical case of a 73-year-old woman with a solitary intramedullary spinal cord metastasis as the initial manifestation of a carcinoid type of tumour. The patient was admitted to our department while presenting a rapid onset of paraparesis. Magnetic resonance imaging was performed, which showed an intramedullary mass at the C2–C6 vertebral level with a heterogeneous contrast enhancement. In light of these findings, the patient underwent surgery for a partial tumour resection. The lesion resulted in being a small-cell neuroendocrine type of carcinoma. This peculiar type of tumour presents similar radiological characteristics to the anaplastic astrocytoma type, which is why our diagnostical mismatch occurred. This is the report of a rare case of solitary intramedullary spinal cord metastasis, which is the result of an initial presentation of a lung small-cell neuroendocrine type of carcinoma. We conclude that ISCMs should be regularly considered as a part of the differential diagnosis of intramedullary lesions, especially in the case of a rapid onset and deterioration of neurological symptoms.

## 1. Background

Based on “The 2015 World Health Organization Classification of Lung Tumors”, pulmonary neuroendocrine tumours are considered as part of epithelial tumours and can be further subclassified into the subtypes of small-cell carcinoma, which are the most common [1]. In the United States, the incidence rate of pulmonary neuroendocrine tumours is 5.25/100,000 [2]. It is, therefore, a rare neoformation that consists of approximately 1% of all pulmonary tumours. Generally, the gold-standard therapy for patients presenting lung tumour neoformation is surgical intervention. However, this is different when addressing metastatic occurrence, as there is no clearly defined therapeutic approach in this case [3]. Intramedullary spinal cord metastases (ISCMs) account for 4.2 to 8.5% of all central nervous system (CNS) metastases, nearly 3.5% of all spinal metastases, 1–3% of all intramedullary tumours, and 0.6% of all spinal cord tumours [2,4,5]. Their incidence rate is estimated to be between 0.9 and 2.1% in the autopsies of cancer patients [3,6]. Lung (48%) and breast (16%) tumours are the most common primary tumours for ISCMs [7]. Lung neuroendocrine tumours (NETs) account for approximately 1 to 2% of all lung tumours in adults and 20 to 30% of all NETs [8,9]. Metastases to the spine and spinal cord are uncommon, and intramedullary-type metastasis is rare [7].

Primary astrocytoma of the spinal cord is a subtype of glioma, which most frequently presents as an intramedullary spinal cord cancer [6]. Its incidence rate accounts for 4.8 per million per year. It is characterised by its slow growth; it presents cystic areas (usually intratumoural) and appears as a well-circumscribed lesion [7]. Primary astrocytoma is frequent in the age group of 35–59-year-olds. It is slightly more frequent in men than in women. Potential risk factors include genetic causes (some genes have shown to be correlated to this pathology), atopic illnesses, and exposure to ionising irradiation [1,10,11].

On magnetic resonance images, astrocytoma typically appears as isointense or hypointense in T1weighted sequences, and hyperintense in those that are T2 weighted. At the post-contrast enhancement phase, astrocytoma often shows a minor enhancement; cystic formations show peripheral enhancement [6,8]. Syringomyelia is a common finding in intramedullary spinal cord tumours, commonly located at the caudal and cranial borders of the lesion. However, radiological examination of syringomyelic lesions does not show any enhancement on MR images [11]. 

We report on the clinical case of a 73-year-old woman with solitary ISCM as the first manifestation of a lung small-cell neuroendocrine carcinoma. In addition, we performed a literature review of cases with a similar presentation which was elaborated further.

## 2. Case Presentation

### 2.1. Patient History and Examination

A 73 year-old-woman was presented at our department with a 2-month history of severe neck pain and a chief complaint of numbness in her arms. A cervical X-ray was previously performed, which resulted in no anomalies. The primary care provider advised her to have physiotherapy. Due to the worsening of the pain and complaints of weakness in her legs, she was admitted to the emergency department. Neurologic examination revealed a spastic paraparesis and numbness in all four limbs. A cranial nerve objective examination produced normal results. She was able to walk, with some difficulty, for short distances. In addition, her medical history reported urge incontinence. A general physical examination did not reveal any other signs or symptoms; no other conditions were referred. She confirmed that any history of cancer or smoking were not present.

### 2.2. Neuroimaging

**Figure 1:**
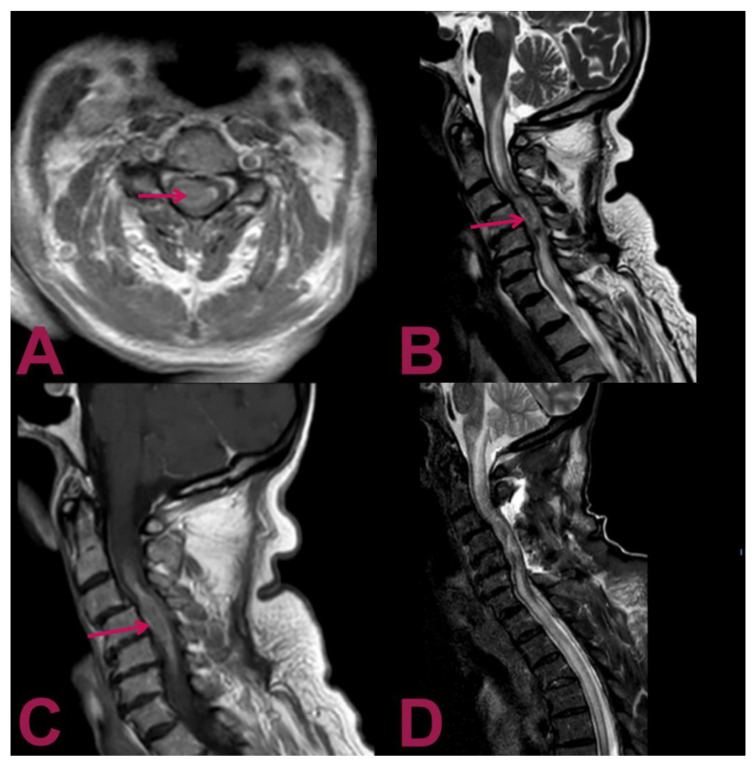
Magnetic resonance imaging (MRI) of the cervical spine. (**A**) Gadolinium-enhanced T1-weighted axial MRI shows an intra-axial lesion containing hemosiderin (arrow) with predominant right paramedian development. (**B**) T2-weighted sagittal MRI shows an extensive syringomyelic cavity, which covers the caudal portion of the floor of the IV ventricle and affects the posterior portion of the bulb–medullar junction extending up to the D5 level inferiorly. The syringomyelic cavity is the result of the newly formed tissue extending from C4 level for about 6 cm with a maximum diameter of about 15 mm (arrow). (**C**) Gadolinium-enhanced T1-weighted sagittal MRI displays the intramedullary mass at the C2–C6 level with a heterogeneous contrast enhancement pattern (arrow). (**D**) Postoperative T2-weighted sagittal MRI reveals the outcomes of hemilaminectomy C3–C4–C5 and cervical myelotomy with partial removal of the lesion; noticeably, despite the invasive nature of the surgical intervention, the previous parameters have not deteriorated; no worsening in the syringomyelic cavity is present, and the peri-wound oedema is not increased.

Magnetic resonance images (MRIs) of the cervical spine showed an intramedullary mass at the level of C2–C6 vertebrae, a heterogeneous hyperintense signal on the T2-weighted sequence, with a heterogeneous pattern of contrast enhancement that extended for about 6 cm associated with a syringomyelic cavity, from the bulbar–medullary junction to the D5 level inferiorly (Figure 1). Considering the correlation between these features and the patient’s history, the neuroradiologist made the diagnosis of anaplastic astrocytoma. The MRI of the brain and the preoperative chest X-ray were normal.

### 2.3. Surgical Procedure

Based on the progressive neurological worsening and MRI images, surgical treatment was indicated. Surgery was performed with the aid of intraoperative neuromonitoring. The patient underwent a C3–C6 laminectomy procedure using a posterior approach. Intraoperatively, the dura appeared as intact. Once exposed to the spinal cord, a midline myelotomy was performed. The tumour appeared to infiltrate the spinal cord without any cleavage plane. The lesion was partially removed, and an extemporaneous biopsy was taken for histologic examination. A mild worsening of the intraoperative motor evocated potentials (MEPs) occurred during the procedure.

### 2.4. Histologic Examination

The histological analysis showed nervous tissue infiltration by poorly differentiated malignant carcinoma cells. These consist of epitheliomorphic cells, which are characterised by poor cytoplasm and medium-sized nuclei. In addition, frequent cellular spindling was present, as well as nuclear moulding and necrosis. The histologic examination was confirmed by immunohistochemistry, which resulted in CKAE1AE3++/CK7+/GFAP-/pS100-/HMB45-/TTF1++/chromogranin A + (dots on 60% of cells).

**Figure 2:**
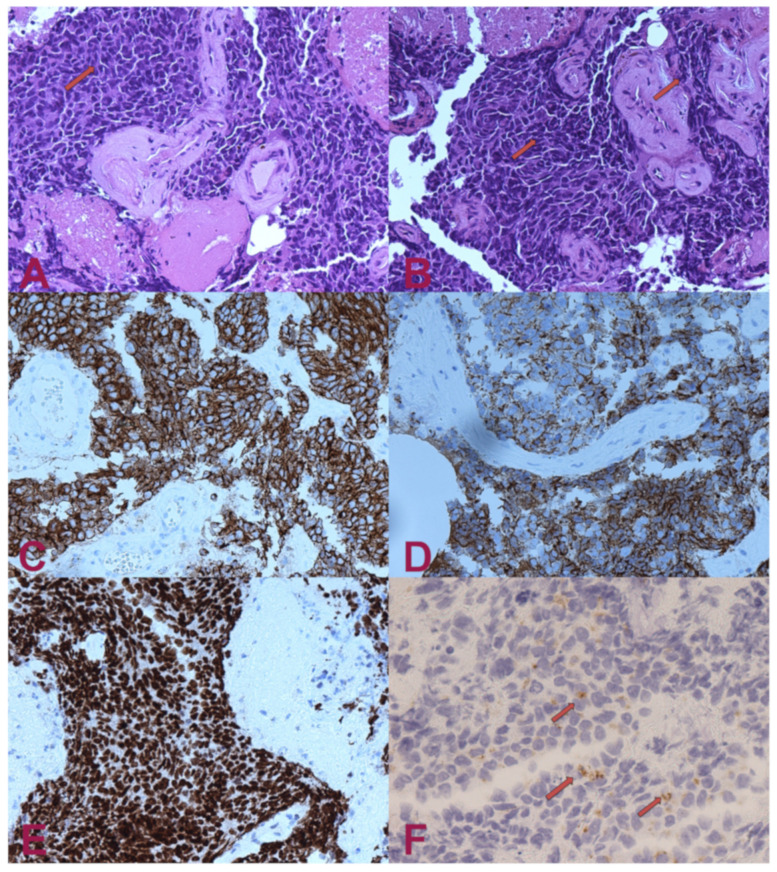
Histologic examination. (**A**) H.E.— small-cell carcinoma infiltrating nervous tissue. The cells have small, intense hyperchromatic nuclei, rounded and polygonal-shaped, and scant cytoplasm. Occasionally, it is possible to note “moulding” signs among the nuclei (arrow). (**B**) H.E.— in some areas, the nuclei have elongated shape (“fusiform”) or squashed (arrows). (**C**) Pan-cytokeratin (clone AE1/AE3)— the cytoplasm of the tumour cells are positive for cytokeratin. Staining for pan-cytokeratin, such as AE1/AE3, helps demonstrate that the tumour is a carcinoma rather than a lymphoid lesion (pan-CK negative and CD45 positive). (**D**) Positivity for cytokeratin 7 (CK7). Small-cell lung carcinoma (SCLC) is usually positive for CK7 and negative for CK20. About half of SCLC stain with CK7 and <10% with CK20. (**E**) Transcriptional Thyroid Factor 1 (TTF1)— strong positivity for TTF1 in almost all neoplastic cells is characteristic, but not specific, for small-cell carcinoma of the lung. Although the stain is less intense, it can be positive in 44–80% of extrapulmonary small-cell carcinomas. (**F**) Chromogranin A— dot-like granular positivity for chromogranin A is a marker of neuroendocrine differentiation of the neoplasm. Up to two-thirds of SCLC are negative for chromogranin and synaptophysin. When these markers are positive, this means that they have high specificity for neuroendocrine differentiation, unlike CD56, which is highly sensitive but not very specific. CD56 will stain approximately 90–100% of cases; however, it is less specific. The interpretation for SCLC diagnosis needs to be performed carefully in the appropriate morphological context and characteristics.

These findings highly suggest the presence of a poorly differentiated lung small-cell neuroendocrine carcinoma. The strong signal for the TTF1 marker is indicative of lung origin. The negativity for the CK20 marker is helpful for the differential diagnosis of the other metastatic small-cell neuroendocrine carcinomas, such as Merkel cell carcinoma, which would result as being positive for CK20 (Figure 2).

### 2.5. Postoperative Course

**Figure 3:**
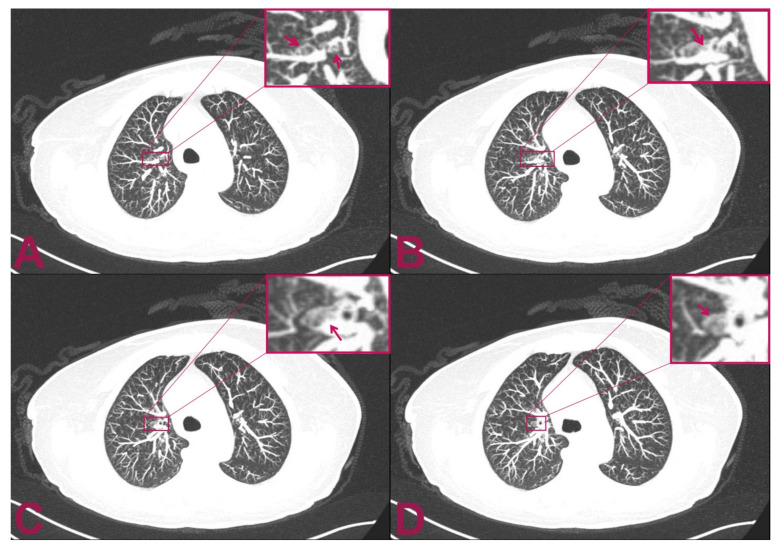
Postoperative multilayer spiral CT scan of the chest with intra-venous administration of contrast medium. The patient is affected by intramedullary cervical metastasis from a primary small-cell neuroendocrine carcinoma of the lung. (**A**,**B**) Axial views show two small, rounded, and confluent nodules located at the level of the superior segment of the right upper lobe of the lung which have a maximum diameter of 9 × 3 mm. (**C**,**D**) The CT axial view shows an additional 2.5 mm abnormal small, solid nodular formation that is located caudally to the previously indicated nodules. The mass appears adherent to the bronchial tube and measures 8 mm in maximum diameter.

The postoperative course was regular, with no additional neurological deficits compared to preoperative. In the following days, we performed a total-body computed tomography (CT) scan that revealed a small, solid mass in the superior segment of the right upper lobe of the lung (Figure 3). Unfortunately, she presented a rapid worsening of her neurological status and prematurely died within three weeks due to a sudden increase in perilesional oedema at the bulb of the spinal cord, which compressed the respiratory centres.

## 3. Discussion

Although NETs can develop anywhere in the body, they usually occur in the gastrointestinal tract (58–75%) and the lung (20–31%) [8,9]. They are considered rare lesions, occurring in only 1–2 cases per 100,000, usually metastasising to lymph nodes, the liver, the lungs, and to bones [8]. However, spinal metastases of NETs are extremely rare, occurring in less than 2%, while ISCM of NETs is even rarer [2]. 

Reported incidences of ISCMs are rising, which is probably due to the advances in cancer treatments and the improvement in life expectancy. Moreover, cases of ISCMs exhibiting this type of metastasis as the first clinical manifestation of primary malignant lung cancer are extremely rare [10]. Therefore, these patients have a poor prognosis with a low survival rate, even with advanced oncologic therapy support. In most cases, the main goal is to achieve improved neurologic outcomes and quality of life. Surgery could be an option only in selected patients. 

In addition, a literature review regarding the ISCM of lung neuroendocrine tumours resulted in few cases being found (Table 1) [2,12,13,14,15,16]. In all cases, the primary tumour origin was known. According to our search, this is the second report of an ISCM being the initial presentation of a primary lung small-cell neuroendocrine carcinoma.

This is of particular interest due to the fact that the MRI has characteristics that can mimic the anaplastic astrocytoma and must be taken into consideration as part of a differential diagnosis. It is crucial in patients with the rapid onset of neurological deficits or patients who present risk factors for the development of the primitive tumour, which in rare cases can metastasise in this infrequent location.

## 4. Conclusions

We reported a rare case of an intramedullary spinal cord metastasis, presented as the first manifestation of a lung small-cell neuroendocrine carcinoma. Additionally, this is the first case reported in the literature of solitary intramedullary metastasis of the cervical segment originating from lung cancer. As we noticed, based on the magnetic resonance imaging for an intramedullary infiltrating lesion, the diagnosis is likely compatible with an intramedullary astrocytoma. The lesion presented an intense and uneven post-contrast enhancement signal. Cranially and caudally to the pathological tissue, there was a syringomyelic cavity. These characteristics can likely mislead to the diagnosis of primitive intramedullary lesions such as diffuse astrocytoma.

However, in cases of the rapid onset of neurological symptoms or in patients who carry known risk factors for developing primary lung tumours, it is rather important to consider intramedullary metastasis as a differential diagnosis. In suspected cases, performing a total-body CT scan can be advantageous to exclude a primary tumour elsewhere or to find other metastases suggestive of the secondary lesion. This would allow the surgeon to make the correct diagnosis without proceeding to the myelotomy procedure to obtain the biopsy sample for analysis, thus exposing the patient to the unnecessary risks of undergoing the open neurosurgical intervention.

## Figures and Tables

**Table 1:** Reported cases of intramedullary spinal cord metastasis from neuroendocrine lung tumours (NETs).

Author, Year	Age/Sex	Localisation	Histology	Initial Presentation	Surgery
Li et al., 2010 [12]	33/F	Multiple	Atypical carcinoid	No	Yes
Duransoy Y.K. et al., 2012 [13]	74/F	T9–T10	SCLC	Yes	Yes
Kumar et al., 2015 [14]	57/M	T11–T12	Atypical carcinoid	No	Yes
Payer et al., 2015 [2]	64/F 34/F	T5–T6 T9	Neuroendocrine tumour of the lung	No	Yes
Kalkan et al., 2016 [15]	50/F	Multiple	Neuroendocrine tumour of the lung	No	N/A
Osawa et al., 2018 [16]	70/M	T11	SCLC	No	No
Presented case	73/F	C2–C6	Neuroendocrine tumour of the lung	Yes	Yes

SCLC—small-cell lung carcinoma, F—female, M—male, N/A—not available.

## Abbreviations

ISCM	Intramedullary spinal cord metastasis
C.T	Computed tomography
MRI	Magnetic Resonance Imaging
SCLC	Small-cell lung cancer
CKAE1AE3	Cytokeratin AE1 / AE3
CK7	Cytokeratin 7
GFAP	Glial fibrillary acidic protein
pS100	S100 proteins
HMB45	Human Melanoma Black 45
TTF1	Transcriptional thyroid factor 1
